# Smart luminescent nanoclusters with dynamic covalent bond for reversible information encryption

**DOI:** 10.1002/smo.20240053

**Published:** 2024-12-10

**Authors:** Bin Bin Chen, Ya Ting Gao, Meng Li Liu, Da Wei Li, Qian Liu, Zheng Zhao, Ben Zhong Tang

**Affiliations:** ^1^ School of Science and Engineering Shenzhen Institute of Aggregate Science and Technology The Chinese University of Hong Kong, Shenzhen (CUHK‐Shenzhen) Shenzhen City Guangdong China; ^2^ School of Chemistry and Molecular Engineering Key Laboratory for Advanced Materials Shanghai Key Laboratory of Functional Materials Chemistry Feringa Nobel Prize Scientist Joint Research Center Frontiers Science Center for Materiobiology and Dynamic Chemistry East China University of Science and Technology Shanghai China; ^3^ Department of Urology Tianjin First Central Hospital Tianjin China

**Keywords:** dynamic covalent bond, dynamic structural change, luminescent nanoclusters, reversible information encryption, Schiff base crosslinking

## Abstract

Luminescent nanoclusters (NCs) have attracted much attention because of their superior photophysical properties; however, the design of dynamic NCs with reversible structural change is highly challenging. Herein, we synthesize a kind of dynamic luminescent NCs through Schiff base crosslinking between triethylenetetramine (TETA) and tannic acid at room temperature. The proposed NCs have an excitation‐independent blue emission, and the maximum emission is available at about 458 nm with two excitation centers. Furthermore, the crosslinking degree of the NCs can be effectively adjusted by TETA and their formation is a kinetic‐control process. Most importantly, the proposed NCs show a property of pH‐controlled reversible depolymerization and polymerization, accompanied by a cyclic “on‐off‐on” photoswitching, which is directly attributed to pH‐stimulated reversible C=N bond cleavage and re‐formation. Because of the reversible structure change properties, the dynamic NCs have been well used in reversible information encryption. This new finding provides not only us with a powerful strategy to study the structure–properties relationship of luminescent NCs but also a design idea for constructing smart optical nanomaterials.

## Graphical abstract

The dynamic luminescent nanoclusters (NCs) with reversible structural change are constructed by a facile Schiff base crosslinking. The proposed NCs exhibit a unique property of pH‐controlled reversible depolymerization and polymerization, accompanied by a cyclic “on‐off‐on” photoswitching, which is directly attributed to pH‐stimulated reversible C=N bond cleavage and re‐formation.

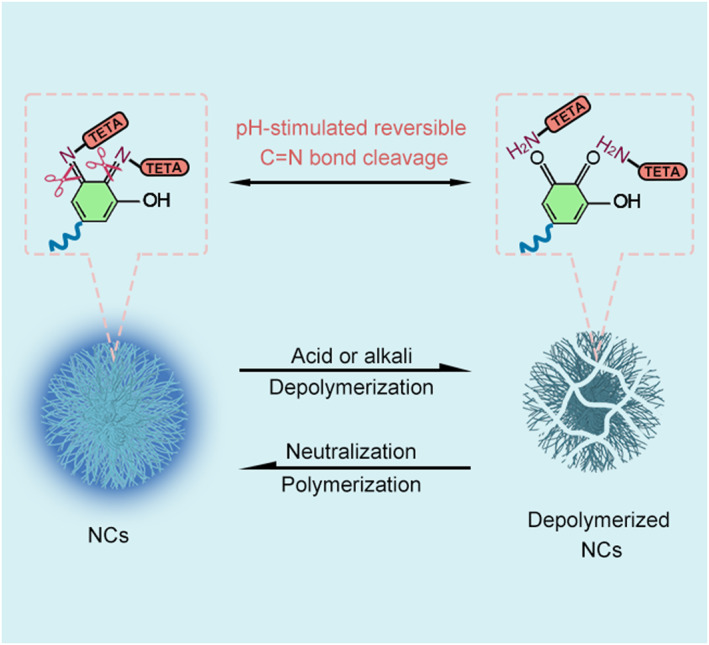

## 1 INTRODUCTION

Dynamically reversible luminescent materials with stimulus responsiveness have recently received considerable attention because of their unique optical properties, which have been widely used for numerous fields, such as chemo/biosensing, information encryption, bioimaging, and disease diagnosis.[^1^, ^2^, ^3^, ^4^, ^5^, ^6^, ^7^] Currently, luminescent materials with reversible photoswitching are mainly obtained by the structural design at the molecular level;[^8^, ^9^, ^10^] however, most organic molecular species suffer from the problems of easy photobleaching and poor water solubility. As a comparison, organic luminescent nanomaterials possess the improved photostability and enhanced water solubility,[^11^, ^12^, ^13^, ^14^, ^15^, ^16^] showing a better application prospect.

Dynamic organic luminescent nanomaterials with reversible structural change have rarely been reported so far. The main reason is that the chemical bonds of organic luminescent nanomaterials are mostly irreversible, in particular in the aggregate state. Worse still, traditional organic luminescent molecules often encounter aggregation‐caused quenching phenomenon when aggregating into nanoparticles by the embedding method.[^17^, ^18^] Meanwhile, typical organic luminescent materials are generally based on π‐conjugated system, which may have biotoxicity and are difficult to degrade, limiting their applications in living system and causing pressure to ecological environment.^[19]^

Recently, natural nonconjugated biomolecules have been regarded as promising molecular precursors for synthesizing organic luminescent materials because of their wide range of sources, low cost, and biocompatibility.[^20^, ^21^, ^22^] Although the molecular species of these nonconjugated structures are nonluminescent or weakly emissive due to the absence of intrinsic chromophores, they will display strong visible emission once they are clustered to form aggregates.[^23^, ^24^] While the irreversible bonding of luminescent aggregates makes it difficult to regulate their optical properties, further hindering the exploration of optical mechanism. Therefore, the construction of dynamic luminescent nanomaterials with reversible structural changes is of great significance not only for the in‐depth research on the structure–property relationship of luminescent nanomaterials but also for the development of smart optical nanomaterials.

**Scheme 1:**
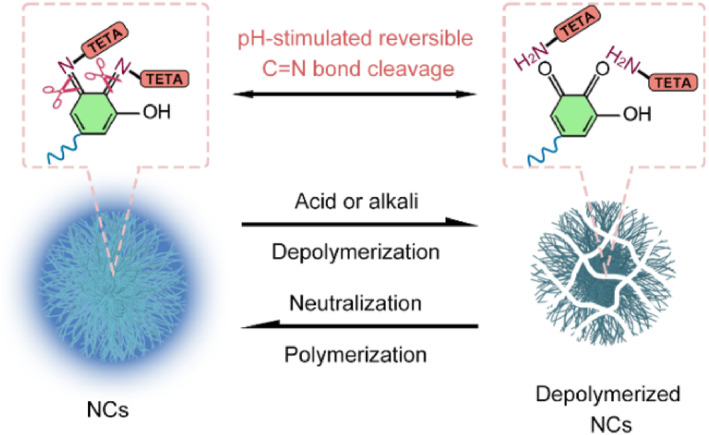
Schematic illustration of the reversible depolymerization and polymerization of the nanoclusters based on the acid/alkali‐controlled reversible C=N bond cleavage and re‐formation.

Dynamic covalent bond is a reversible chemical bond, which provides a powerful tool for constructing dynamic luminescent nanomaterials based on its characteristics of dissociation and re‐formation under specific stimulation. Inspired by this, in this work, we design and prepare blue emissive nanoclusters (NCs) by the Schiff base reaction using weakly emissive tannic acid (TA) and nonluminescent triethylenetetramine (TETA) as nonconjugated molecular precursors. The proposed NCs have a maximum emission of about 458 nm with two excitation centers. The crosslinking degree of the NCs is TETA‐dependent, and the larger the crosslinking degree, the higher the luminescent efficiency. Most importantly, NCs have a unique property of dynamically reversible structural change under pH stimulation based on acid/alkali‐controlled C=N bond cleavage and re‐formation (Scheme 1). The pH‐induced reversible depolymerization and polymerization can cause a cyclic “on‐off‐on” photoswitching, showing a promising application in reversible information encryption.

## 2 RESULTS AND DISCUSSION

### 2.1 Preparation and characterization of the nanoclusters

**FIGURE 1:**
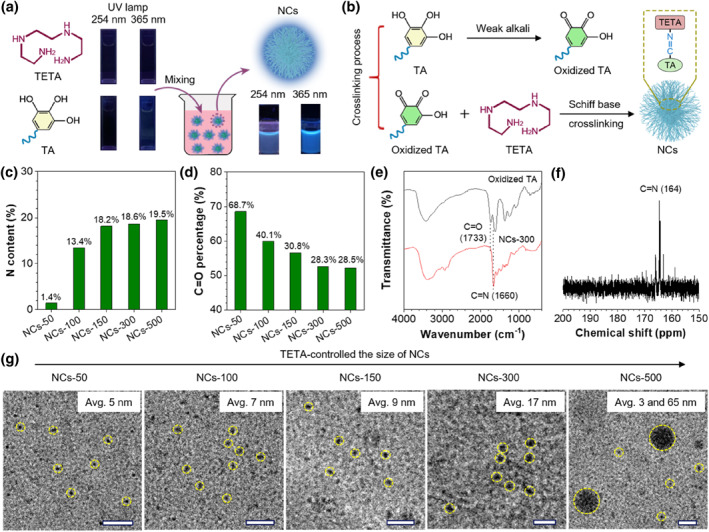
Synthesis and structural analysis of NCs. (a) Illustration of the synthesis process of NCs. (b) Main chemical reactions in the synthesis of NCs. (c) Nitrogen content of five NCs measured by the X‐ray photoelectron spectrometer spectra. (d) The percentage of C=O bond of five NCs based on the high‐resolution O1s spectra. (e) Fourier transform infrared spectra of oxidized and NCs‐300. (f) ^13^C nuclear magnetic resonance spectrum of NCs‐300. (g) High‐resolution transmission electron microscopy images of five NCs. Scale bar: 40 nm. NCs, nanoclusters.

A facile Schiff base crosslinking strategy is developed for the preparation of dynamic luminescent NCs at room temperature. As shown in Figure 1a, weakly emissive TA and nonluminescent TETA can be polymerized into NCs with bright blue fluorescence (FL). The formation mechanism of NCs was first explored (Figure 1b). It is widely known that TA molecules with rich phenolic hydroxyl groups (Supporting Information S1: Figure S1) can be rapidly oxidized into *α*‐hydroxy‐*ortho*‐quinone species under weak alkaline conditions caused by the TETA.^[25]^ Subsequently, the C=O groups of the *ortho*‐quinone can react with the NH_2_ groups of TETA by Schiff base reaction to form the imine structure (C=N bond). Meanwhile, TETA can function as the crosslinker to connect *ortho*‐quinone molecules to form an extended polymer chain, which further aggregate to in situ generate NCs by either covalent or noncovalent bonding.[^26^, ^27^]

Five kinds of NCs were synthesized by changing the TETA volume used from 50 to 500 μL (Supporting Information S1: Table S1), which were named sequentially as NCs‐50, NCs‐100, NCs‐150, NCs‐300, and NCs‐500. A large intensity ratio (*I*_*D*_/*I*_*G*_ = 1.11) value of the *D* band (disorder) to the *G* band (crystalline) in the Raman spectrum suggests that the formed NCs have an amorphous structure (Supporting Information S1: Figure S2).^[28]^ X‐ray photoelectron spectrometer (XPS) and Fourier transform infrared (FT‐IR) techniques were used to investigate the structure of NCs. Based on the XPS N1s spectra (Figure 1c and Supporting Information S1: Figure S3, Supporting Information), the nitrogen content of NCs is sharply increased from 1.4% (NCs‐50) to 19.5% (NCs‐500). Conversely, the percentage of C=O bond in XPS O1s spectra is greatly decreased from 68.7% (NCs‐50) to 28.5% (NCs‐500) (Figure 1d and Supporting Information S1: Figure S4).

These results indicated that C=O was efficiently converted as a C=N bond during the formation of the NCs. The nitrogen content and the percentage of the C=O bond reached a plateau when the volume of TETA is 300 μL, indicating that the C=O bond was almost completely converted to the C=N bond in NCs‐300; thus NCs‐300 was chosen for the following experiment. FT‐IR spectra (Figure 1e) further confirmed the structural evolution of NCs. Strong stretching vibration of the C=O bond at 1733 cm^−1^ is identified in the oxidized TA. This peak is replaced with the C=N stretching at 1660 cm^−1^ in NCs‐300,^[29]^ indicating the formation of C=N bond by Schiff base condensation at the cost of C=O. Moreover, the peak at about 164 ppm in ^13^C nuclear magnetic resonance spectrum of NCs‐300 is ascribed to the carbon in the C=N,^[30]^ because the peak of carbonyl carbon is higher than 170 ppm (Figure 1f).^[31]^ These results revealed that the proposed NCs are formed by Schiff base crosslinking, which contain numerous dynamic C=N bonds.

As a crosslinker, TETA can also tune the crosslinking degree of NCs. As shown in Figure 1g and Supporting Information S1: Figure S5, the increase of TETA amount can result in the production of larger sized NCs with the diameter increased from about 5 to 60 nm, which is further confirmed by the hydrodynamic size (Supporting Information S1: Figure S6, Supporting Information). Meanwhile, many ultra‐small NCs of about 3 nm are found in NCs‐500 sample in addition to the large NCs of about 60 nm. This result suggests that the growth of NCs is a kinetic‐control process. More TETA causes faster reaction rate, which will cause a local low concentration of TA, which prohibits the growth of NCs.

### 2.2 Optical properties of the nanoclusters

**FIGURE 2:**
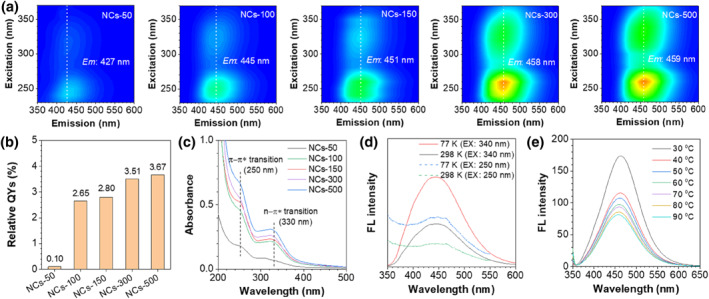
Optical properties of NCs. (a) 3D FL spectra of five NCs with the same concentration. (b) Relative quantum yields of NCs. (c) Absorption spectra of five NCs with the same concentration. (d) FL spectra of NCs‐300 measured at 77 and 298 K. (e) FL spectra of NCs‐300 excited at 340 nm at different temperatures. NCs, nanoclusters.

Subsequently, the optical properties of NCs were investigated. As shown in Figure 2a, all the NCs display excitation‐independent blue emission that can be excited by two different excitation centers (250 and 340 nm). The maximum emission of NCs is gradually red‐shifted from 427 nm (NCs‐50) to 459 nm (NCs‐500), which is attributed to the increased crosslinking degree that can reduce the electronic bandgap, resulting in a red shift in FL.^[32]^ Meanwhile, the relative quantum yields of NCs are obviously increased from 0.1% (NCs‐50) to 3.67% (NCs‐500) (Figure 2b and Supporting Information S1: Figure S7) because the increase of crosslinking degree help rigidify the polymer structure, thereby exhibiting higher fluorescent efficiency. The results of photo‐excitation dynamics showed that the crosslinking degree had little effect on the FL lifetimes of NCs, maintaining around 5 ns **(**Supporting Information S1: Figure S8).

Absorption spectra displayed that the prepared NCs had two characteristic absorption peaks at 250 and 330 nm (Figure 2c), which were attributed to the π−π* transition and n−π* transition, respectively. As the degree of crosslinking increased from NCs‐50 to NCs‐500, the photoabsorption of NCs was greatly enhanced, causing a great improvement in the efficiency. Variable‐temperature spectra (Figure 2d) further showed that the FL intensity of the NCs obviously increased along with the decrease of temperature from 298 to 77 K. Similarly, the FL intensity of NCs gradually decreased with increasing temperature from 30 to 90°C, whether excited at 250 nm or 340 nm **(**Figure 2e and Supporting Information S1: Figure S9). This is because the decrease in temperature can reduce the molecular motion of NC surface, preventing the non‐radiative quenching, resulting in the enhancement of FL emission.

**FIGURE 3:**
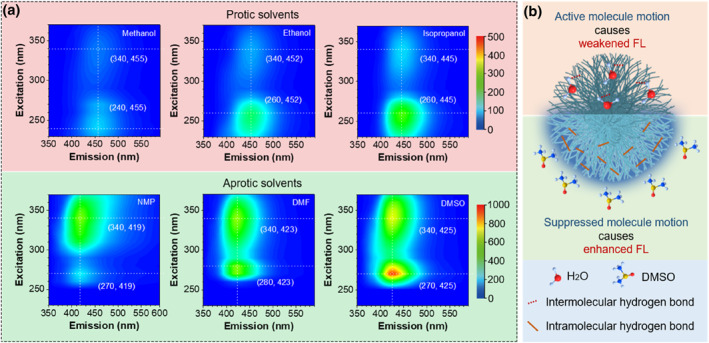
Solvent‐dependent FL of NCs. (a) 3D FL spectra of NCs‐300 in protic and aprotic solvents. (b) Illustration of the effect of solvent molecules on NC FL. Concentration of NCs‐300: 0.01 mg/mL. NCs, nanoclusters.

In order to further elucidate the effect of molecular motion on the FL efficiency of NCs, different organic solvents, including protic and aprotic solvents, were introduced. As shown in Figure 3a, the NCs showed weak FL in protic solvents such as methanol, ethanol, and isopropanol, and their intensities were similar to that of NCs in aqueous solution. The NCs emitted strong blue FL in aprotic solvents such as N‐methylpyrrolidone, N,N‐dimethylformamide (DMF), and dimethyl sulfoxide (DMSO) compared with protic solvents. Compared with protic solvents, the FL intensity of NCs in aprotic solvents can be increased up to 25.5 times (Supporting Information S1: Figure S10). The regulation of FL efficiency of NCs by solvents should be attributed to the regulation of hydrogen bonding on the surface of NCs by solvent molecules (Figure 3b). Because the surface of NCs is rich in hydrophilic functional groups, which can form intramolecular hydrogen bonds in aprotic solvents, thereby enhancing the FL efficiency by rigidifying chemical groups and inhibiting molecular motion. In contrast, the intramolecular hydrogen bonds of surface groups of NCs can be disrupted in protic solvents because protic solvents such as water molecules can form intermolecular hydrogen bonds with surface groups of NCs. This intensified the molecular motion of the NC surface, thereby weakening their FL efficiency. In addition, the lifetime of NCs in DMSO was obviously higher than that of NCs in aqueous solution (Supporting Information S1: Figure S11), strongly indicating that NCs have a more rigid structure in DMSO compared with in aqueous solution.[^33^, ^34^]

### 2.3 Reversible polymerization of the nanoclusters

**FIGURE 4:**
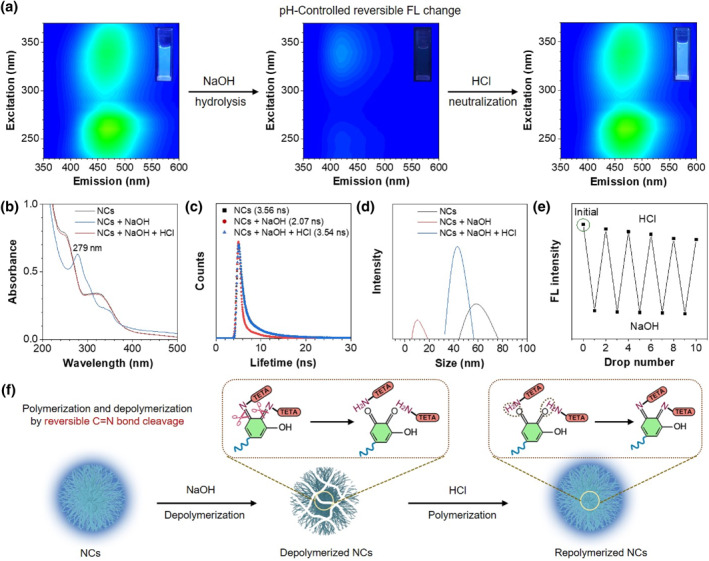
pH‐controlled reversible structural change of NCs. (a) Reversible changes in pH‐adjusted FL of NCs‐300. (b) Reversible absorption spectra, (c) reversible FL lifetime, and (d) reversible size change of NCs‐300 when adding NaOH solution (1 M) and HCl solution (1 M) in turn. (e) Acid/alkali‐controlled reversible FL intensity change of NCs‐300. EX: 340 nm. (f) Mechanism illustration of reversible change of NC structure. Concentration of NCs‐300: 0.01 mg/mL. NCs, nanoclusters.

Since the NCs were prepared by the dynamic C=N bond crosslinking, the structural reversibility of NCs was extensively investigated. The stability of NCs is first studied, and the FL intensity of NCs decreases slightly in the initial stage and then remains constant as the storage time increases **(**Supporting Information S1: Figure S12), indicating that NCs will not self‐degrade in aqueous solution. Moreover, the obtained NCs display a strong photobleaching resistance, and their FL intensity is almost unchanged under 60 min of illumination (Supporting Information S1: Figure S13). Meanwhile, the FL of NCs at different pH solutions was further explored. Results show that the FL efficiency of NCs can be greatly improved under acid solution; conversely, their FL intensity can be decreased with the increase in NaOH concentrations (Supporting Information S1: Figure S14). The significantly enhanced FL in appropriate acidic solutions should be attributed to strong protonation.^[35]^ From the FL intensity of NCs at different pH solutions, when using NaOH solutions with concentrations higher than 100 mM (pH = 13), NCs can be rapidly depolymerized. In light of this, 1 M of NaOH solution is used to regulate the depolymerization of NCs. As shown in Figure 4a, the NCs showed strong blue emission, but their FL can be significantly quenched after the addition of NaOH. Surprisingly, after neutralization with HCl, the FL of the NCs returned to their original state, achieving the pH‐controlled reversible FL change (Supporting Information S1: Figure S15). Absorption spectra were used to investigate the mechanism of FL change (Figure 4b). After the addition of NaOH, the two absorption peaks (250 and 330 nm) of NCs disappeared, and instead, a new peak at 279 nm appeared. This new absorption peak was similar to that of TA or oxidized TA (Supporting Information S1: Figure S16), indicating that NCs underwent an alkaline hydrolysis of the C=N bond. After neutralization with HCl, the absorption band returned to its original state, indicating the re‐polymerization of depolymerized NCs. The lifetime results of first reducing and then recovering further indicated the reversible depolymerization and polymerization process of NCs stimulated by acid/alkali (Figure 4c).

Meanwhile, the results of hydrodynamic size showed that the size of the NCs was reduced to about 12 nm after NaOH treatment, which increased to about 45 nm once the solution was neutralized (Figure 4d), strongly confirming the pH‐adjusted reversible depolymerization and polymerization of the NCs. Furthermore, the repeating stability indicated that the NCs had superior reversible structural change ability even after multiple cycles of depolymerization and polymerization (Figure 4e). Taken together, the reversible depolymerization and polymerization of NCs can be achieved by the pH‐controlled reversible C=N bond cleavage and re‐formation (Figure 4f). Meanwhile, the characteristics of FL quenching when depolymerizing and FL enhancement when polymerizing clearly confirmed that the NCs have a clusterization‐triggered emission property.

### 2.4 Reversible information encryption of the nanoclusters

**FIGURE 5:**
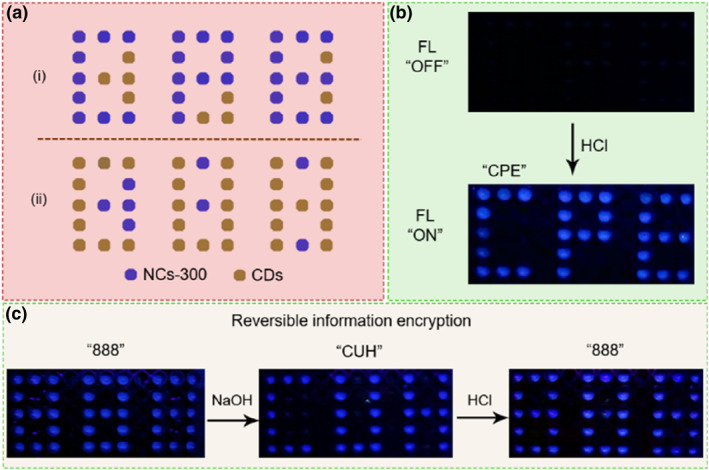
Reversible information encryption of the NCs. (a) The designed information encryption pattern using NCs‐300 and alkali‐insensitive CDs. (b) Schematic illustration of information encryption with the NCs‐300 and CDs (0.1 μg/mL). (c) Schematic illustration of reversible information encryption with the NCs‐300 and CDs (1 mg/mL). CDs, carbon dots; NCs, nanoclusters.

Because of their unique structural properties, the prepared NCs have been used for the pH‐stimulated information encryption. Carbon dots (CDs) with stable blue FL in NaOH solution were used to assist in designing code numbers (Figure 5a).^[36]^ An acid‐stimulated “off‐on” information encryption mode was developed. As shown in Figure 5b, NaOH solution (1 M) was first mixed with the NCs and CDs, respectively. The blue FL of NCs can be quenched due to the NaOH‐induced depolymerization. Although the FL of CDs cannot be quenched by NaOH, their low concentration (0.1 μg/mL) made the FL of CDs invisible under UV lamp. In view of this, the encrypted information was invisible when excited by 365 nm UV light. However, after neutralization with HCl, the FL of NCs can be recovered but the FL of CDs remained unchanged; thus, a blue “CPE” pattern appeared.

Furthermore, a pH‐stimulated reversible information encryption mode was developed. As shown in Figure 5c, by increasing the concentration (1 mg/mL) of CDs to make their FL intensity consistent with that of the NCs. At this time, a blue “888” pattern was visible under the irradiation of 365 nm UV light. After NaOH treatment, the FL of NCs was immediately quenched and the FL of CDs remained unchanged; thus, the “888” pattern was transformed into “CUH” pattern. Further HCl neutralization resulted in FL recovery of NCs, and the encrypted information was restored to “888” pattern. Therefore, the dynamic luminescent NCs showed the potential applications in reversible information encryption.

## 3 CONCLUSION

In conclusion, we have developed a kind of dynamic luminescent NC based on the Schiff base crosslinking strategy. The prepared NCs had an excitation‐independent blue emission and a TETA‐controlled size property. The greater the degree of crosslinking, the higher the luminescent efficiency. Because of the pH‐controlled reversible C=N bond cleavage and re‐formation, the NCs showed an acid/alkali‐controlled dynamic depolymerization and polymerization process. Based on the characteristics of dynamic structural change, the prepared NCs can be used for the pH‐stimulated reversible information encryption. This work not only deepened our understanding of the optical mechanism of NCs but also constructed smart NCs with dynamic structural changes.

## 4 EXPERIMENTAL SECTION

### 4.1 Reagent and apparatus

NaOH and TA were purchased from Aladdin Reagent Co., Ltd (Shanghai, China). TETA was obtained from Shanghai Titan Scientific Co., Ltd. HCl was obtained from Sigma‐Aldrich Co., Ltd. Dialysis membrane (100−500 Da) for the purification of NCs is commercially available from Solarbio Co., Ltd (Beijing, China). All solutions were prepared using 18.2 MΩ.cm ultrapure water (Millipore‐Q, Shanghai). FL and absorption spectra of NCs were recorded on an LS‐55 Lumine and an Agilent Cary 60 UV‐Vis spectrophotometer, respectively. The lifetimes of NCs are measured using a FLS1000 FL spectrometer. The elemental composition of NCs was measured using an ESCALAB 250Xi XPS. Fourier transform infrared spectra of NCs were determined on a FTIR‐8400S FT‐IR spectrometer. Transmission electron microscopy and high‐resolution transmission electron microscopy images of NCs were performed on a Talos L120C transmission electron microscope. The hydrodynamic size of NCs was measured using a Zetasizer Nano‐ZS particle size analyzer.

### 4.2 Room temperature synthesis of NCs

NCs can be facilely synthesized by the room temperature Schiff base reaction. Briefly, 100 mg of TA is dissolved in water, and then TETA is further added into the solution. After 5 days of reaction, NCs are formed. By varying the volume of TETA from 50 to 500 μL, a series of NCs (named them NCs‐50, NCs‐100, NCs‐150, NCs‐300, and NCs‐500) can be obtained, see **Table S1** for details. Subsequently, the NC solution was dried and absolute ethanol was added and allowed to stand overnight. After that, a large number of precipitate produces and is then removed by repeated centrifugation and resuspension. Furthermore, NCs are purified by a dialysis membrane (100−500 Da). Finally, NCs are dried by lyophilization to obtain pure NCs, which is dispersed in water (1 mg/mL) for further use.

### 4.3 Solvent‐dependent FL

During this experiment, 20 μL of NCs‐300 solution (1 mg/mL) is first dried in a vacuum drying oven, and then 2 mL of different organic reagents are added to re‐disperse. Subsequently, the FL spectra of NCs‐300 in different solvents were measured.

### 4.4 Acid/alkali‐controlled reversible FL change of NCs‐300

During this experiment, 20 μL of NCs‐300 solution (1 mg/mL) was mixed with 1980 μL water, then 20 μL of NaOH solution (1 M) and 20 μL of HCl solution (1 M) were added into NCs‐300 solution in turn. The absorption and FL spectra of NCs‐300 after each drop of NaOH and HCl solution were determined.

## CONFLICT OF INTEREST STATEMENT

The authors declare no conflicts of interest.

## ETHICS STATEMENT

No animal or human experiments were involved in this study.

## Supporting information

Supporting Information S1

## Data Availability

All relevant data supporting the results of this study are available in the article and its supplementary information files. Further data are available from the corresponding authors upon request.
