# Supplementary material for: Smart luminescent nanoclusters with dynamic covalent bond for reversible information encryption
Source: Smart Mol. 2024 Dec 10;3(4):e20240053. doi: 10.1002/smo.20240053 (PMC12755220; doi:10.1002/smo.20240053)
Supplement: Supplementary file 1 — Supporting Information S1 [file SMO2-3-e20240053-s001.docx]

Supporting Information

*for*

Smart Luminescent Nanoclusters with Dynamic Covalent Bond for Reversible Information Encryption

Bin Bin Chen, ^[a]^ Ya Ting Gao, ^[b]^ Meng Li Liu, ^[a]^ Da Wei Li, ^[b]^ Qian Liu, ^[c]^ Zheng Zhao*^, [a]^ and Ben Zhong Tang*^, [a]^

*^a^* School of Science and Engineering, Shenzhen Institute of Aggregate Science and Technology, The Chinese University of Hong Kong, Shenzhen (CUHK-Shenzhen), 2001 Longxiang Boulevard, Longgang District, Shenzhen City, Guangdong 518172, China.

*^b^* Key Laboratory for Advanced Materials, Shanghai Key Laboratory of Functional Materials Chemistry, Feringa Nobel Prize Scientist Joint Research Center, Frontiers Science Center for Materiobiology & Dynamic Chemistry, School of Chemistry & Molecular Engineering, East China University of Science and Technology, Shanghai, 200237, China.

*^c^* Department of Urology, Tianjin First Central Hospital, Tianjin 300192, China.

*E-mail: tangbenz@cuhk.edu.cn and zhaozheng@cuhk.edu.cn.

**
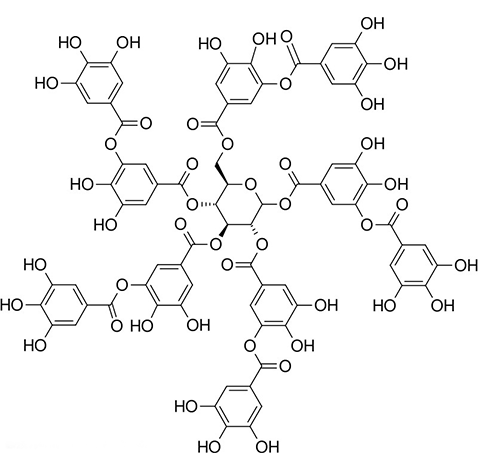
**

**Figure S1.** Chemical structure of TA molecule. TA is a weak acid, which contains five catechol- and pyrogallol-type phenols. These phenolic hydroxyl groups are active, which can take place in a variety of chemical reactions.^[^[^1^](#_ENREF_1)^]^ TA molecule can be rapidly oxidized into the α-hydroxy-*ortho*-quinone species under weak alkaline conditions.^[^[^2^](#_ENREF_2)^]^

**Table S1.** Synthesis of NCs using different volume of TETA. Results show that the content of TETA plays an important role in the formation, structure, and FL properties of NCs. The FL intensity of NCs increases gradually as the increase of the volume of TETA, and the FL intensity of NCs-50 is the weakest. This indicates that TETA can effectively improve the FL efficiency of NCs.

| NCs | TA  (g / μmol) | TETA  (μL / mmol) | Water (mL) | Reaction time (days) | Photos. of NCs ethanol solution | | |
| --- | --- | --- | --- | --- | --- | --- | --- |
|  |  |  |  |  | Visible light | 254 nm UV light | 365 nm  UV light |
| NCs-50 | 0.1 / 58.78 | 50 / 0.34 | 4.95 | 5 | 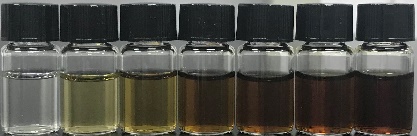 | 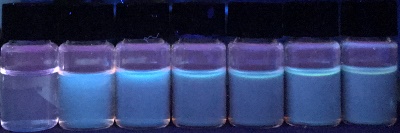 | 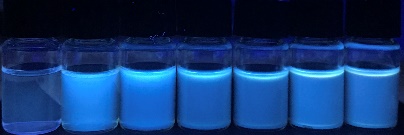 |
| NCs-100 | 0.1 / 58.78 | 100 / 0.67 | 4.9 | 5 | 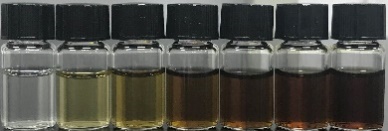 | 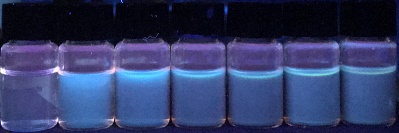 | 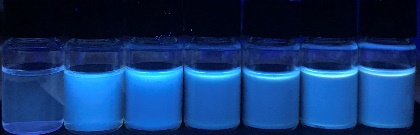 |
| NCs-150 | 0.1 / 58.78 | 150 / 1.01 | 4.85 | 5 | 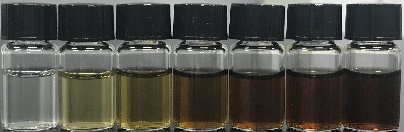 | 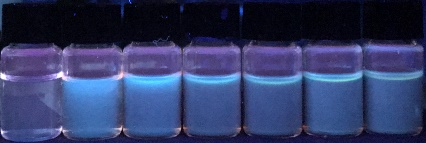 | 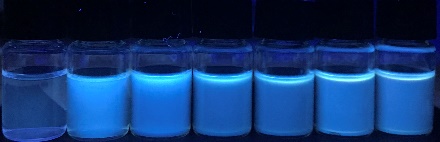 |
| NCs-300 | 0.1 / 58.78 | 300 / 2.01 | 4.7 | 5 | 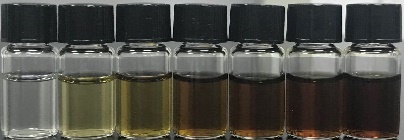 | 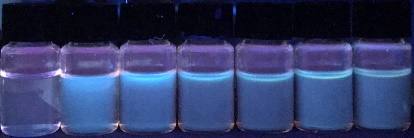 | 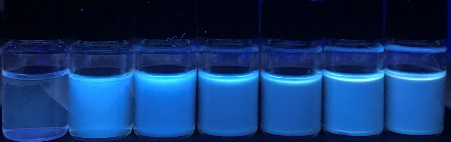 |
| NCs-500 | 0.1 / 58.78 | 500 / 3.36 | 4.5 | 5 | 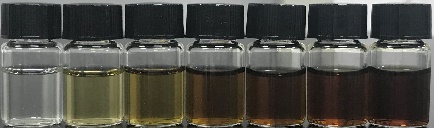 | 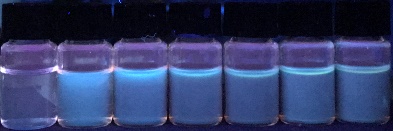 | 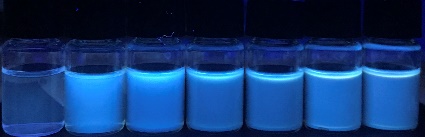 |





**Figure S2.** Raman spectrum of NCs-300. Results show two distinct peaks at about 1370 cm^-1^ and 1560 cm^-1^, which are attributed to the D-band (sp^3^-hybridized) and G-band (sp^2^-hybridized), respectively. A large *I*_D_ / *I*_G_ value of about 1.11 indicates the disordered structure of NCs.


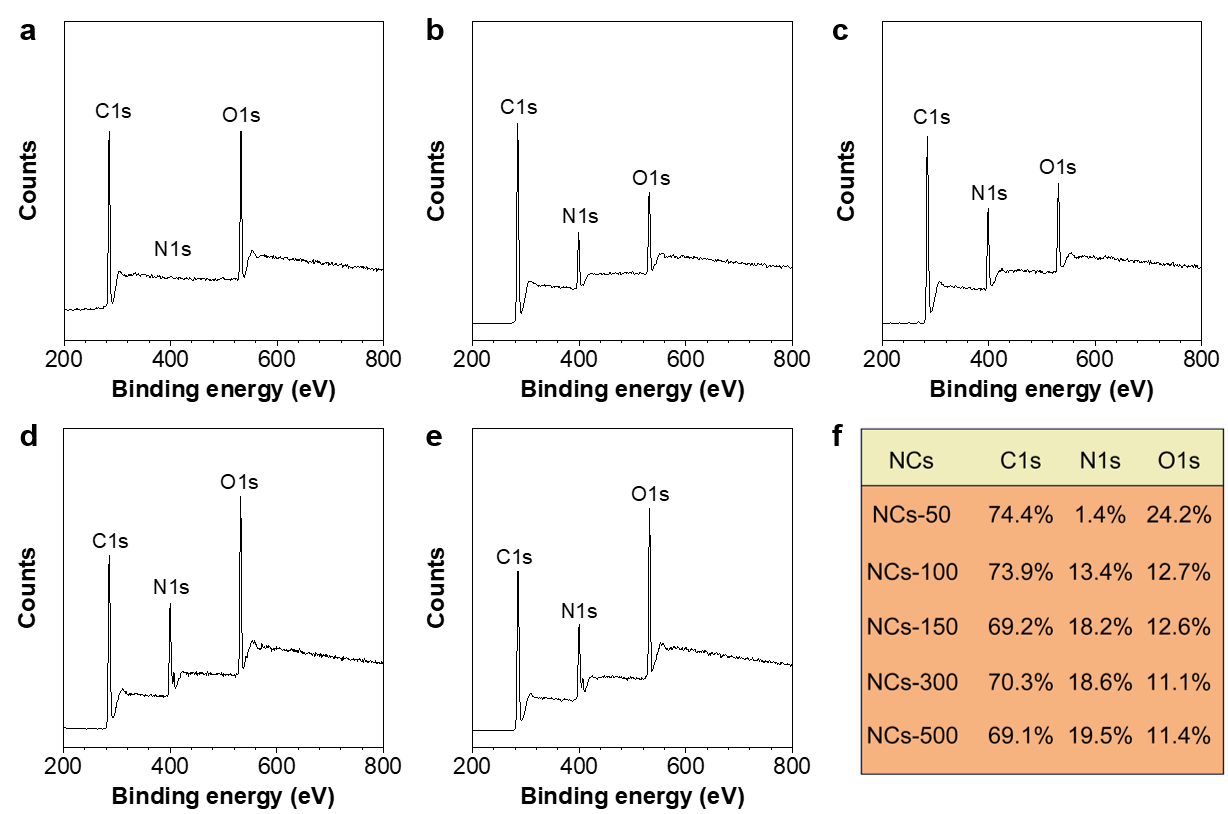


**Figure S3.** XPS spectra of five NCs. XPS spectra of (a) NCs-50, (b) NCs-100, (c) NCs-150, (d) NCs-300, and (e) NCs-500. (f) Atomic percentage of five NCs. Taken overall, the carbon content changes little, and the nitrogen content increases, while the oxygen content decreases from NCs-50 to NCs-500. Compared with NCs-50, the nitrogen content of other NCs greatly increases and the oxygen content greatly decreases, indicating that the conversion of C=O to C=N by Schiff base reaction.


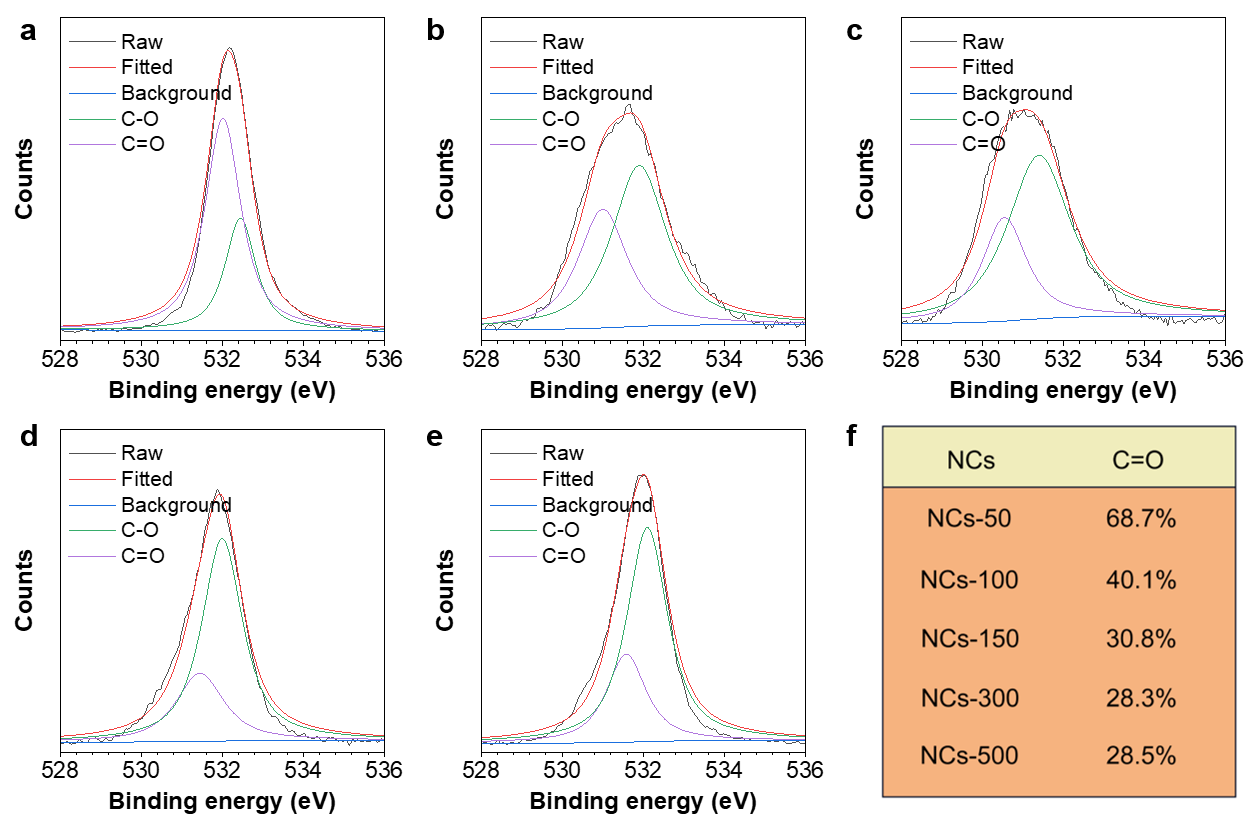


**Figure S4.** High-resolution O1s spectra of five NCs. High-resolution O1s spectra of (a) NCs-50, (b) NCs-100, (c) NCs-150, (d) NCs-300, and (e) NCs-500. (f) Percentage of C=O bond in five NCs. The percentage of C=O bond gradually decreases from 68.7% (NCs-50) to 28.5% (NCs-500), indicating the conversion of C=O to C=N by Schiff base condensation.


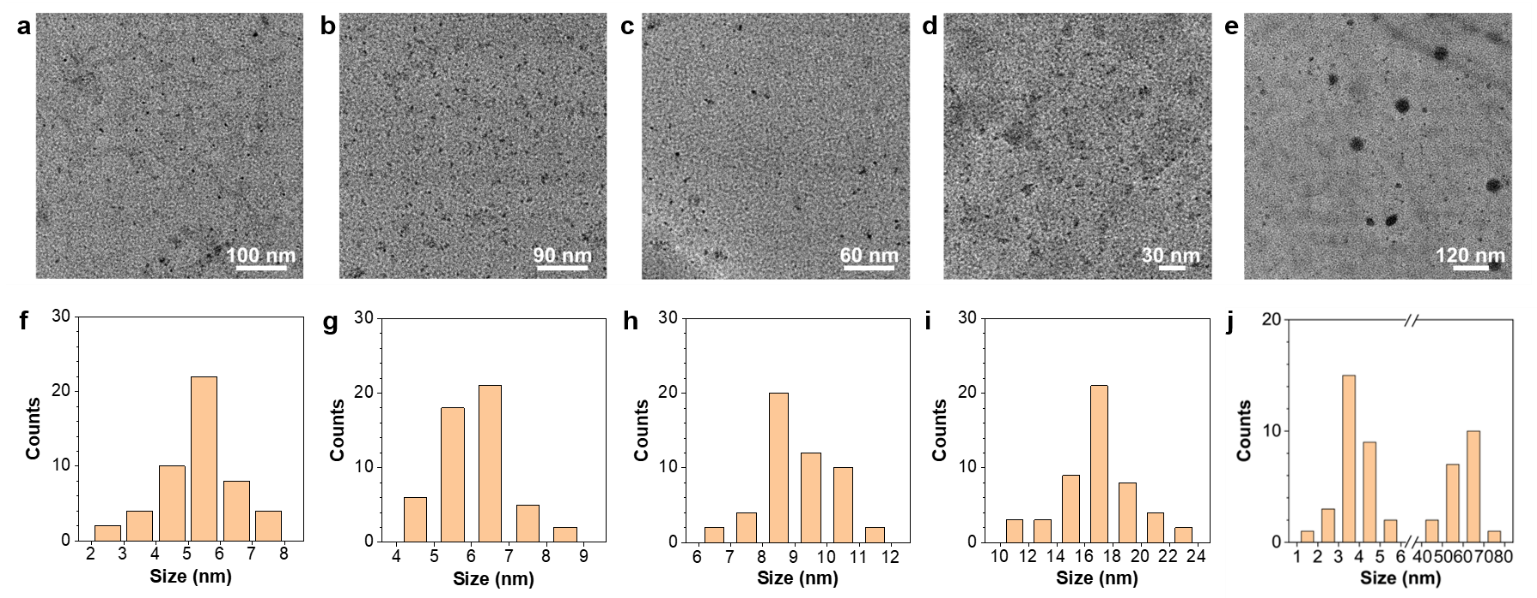


**Figure S5.** TEM images of (a) NCs-50, (b) NCs-100, (c) NCs-150, (d) NCs-300, and (e) NCs -500. Size statistics of (f) NCs-50, (g) NCs-100, (h) NCs-150, (i) NCs-300, and (j) NCs-500. The size of NCs gradually increases from NCs-50 to NCs-500. Moreover, there are two kinds of NCs nanoparticles in the NCs-500 sample solution: the average size of small one is about 3 nm, and the average size of large one is about 60 nm, indicating that the growth of NCs is a kinetic-control process. With increasing TETA, the reaction rate increases. This will cause a local low concentration of TA, which prohibits the NCs growth.


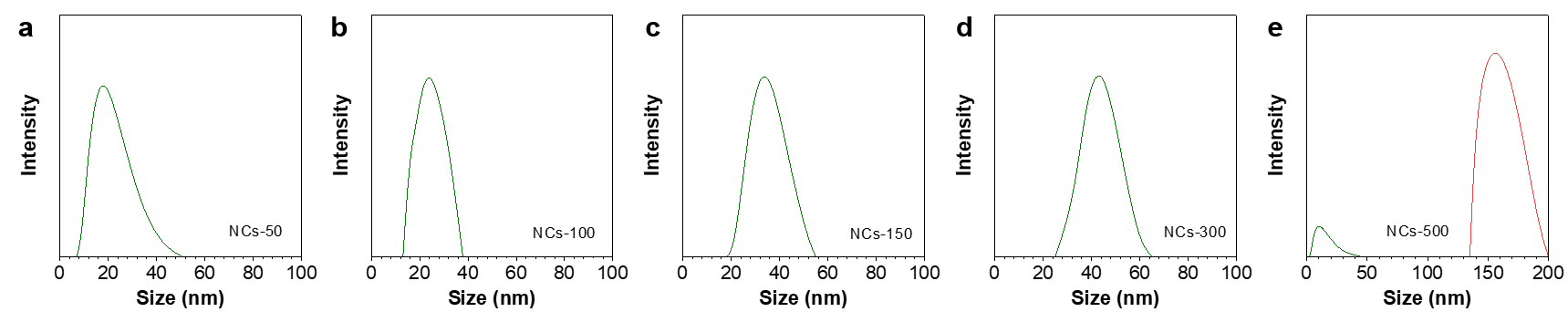


**Figure S6.** Hydrodynamic sizes of (a) NCs-50, (b) NCs-100, (c) NCs-150, (d) NCs-300, and (e) NCs-500. The hydrodynamic size of NCs gradually increases from NCs-50 to NCs-500. For NCs-500 sample, there are two sizes of NCs nanoparticles in the solution: one is about 10 nm, and the other is about 160 nm. This result is consistent with the size obtained by TEM, indicating a kinetic-control process in the synthesis of NCs.


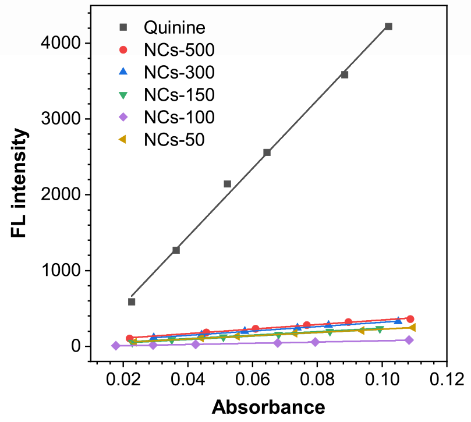


**Figure S7.** Relative QYs of five NCs. Plot of integrated FL intensity vs. absorbance of quinine sulfate and NCs. The relative QYs (Φ) of NCs are calculated using quinine sulfate as a reference. Quinine sulfate (Φ = 0.55) is dissolved in 0.1 M H_2_SO_4_ solution (refractive index (η) = 1.33) and NCs are dissolved in ultra-pure water (η = 1.33). Then QYs of NCs are calculated by comparing the integrated FL intensities (excited at 340 nm for NCs and excited at 360 nm for quinine) and the absorbance values (at 335 nm for NCs and at 360 nm for quinine) of NCs and quinine sulfate. The data is plotted and the slopes of NCs and quinine sulfate are determined. The QY is calculated by the below equation:

Φ_x_ = Φ_Q_ (m_x_ / m_Q_) (η_x_^2^ / η_Q_^2^)

wherein Φ is the QY, m is slope, η is the refractive index of the solvent, Q is the quinine sulfate and x is NCs. The relative QYs of NCs-50, -100, -150, -300, and -500 are 0.1%, 2.65%, 2.8%, 3.51%, and 3.67%, respectively. Results show that the increase of crosslinking degree significantly improves the fluorescent efficiency of NCs.


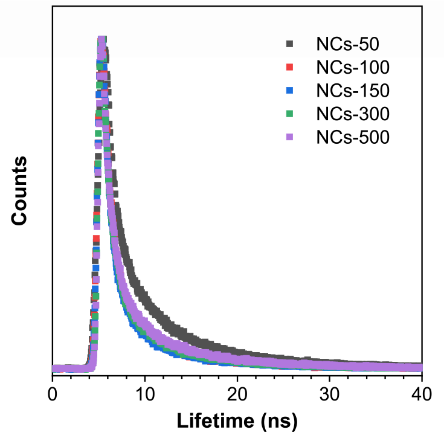


**Figure S8.** FL lifetimes of NCs measured by 375 nm laser. The FL lifetimes of NCs-50, -100, -150, -300, and -500 are 5.77 ns, 4.20 ns, 4.18 ns, 4.55 ns, and 5.08 ns, respectively. Results show that the crosslinking degree has little effect on the FL lifetimes of NCs, maintaining around 5 ns.


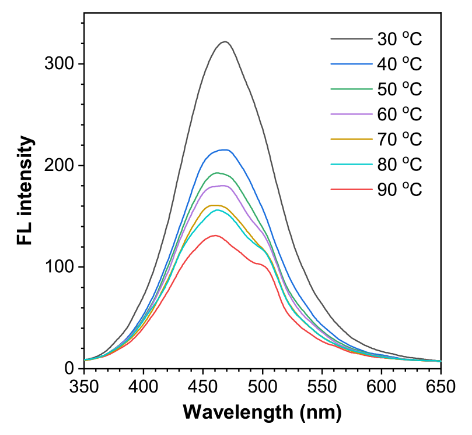


**Figure S9.** FL spectra of NCs-300 excited at 250 nm at different temperatures. Results show that the FL intensity of NCs-300 gradually decreases with increasing temperature from 30 ^o^C to 90 ^o^C.


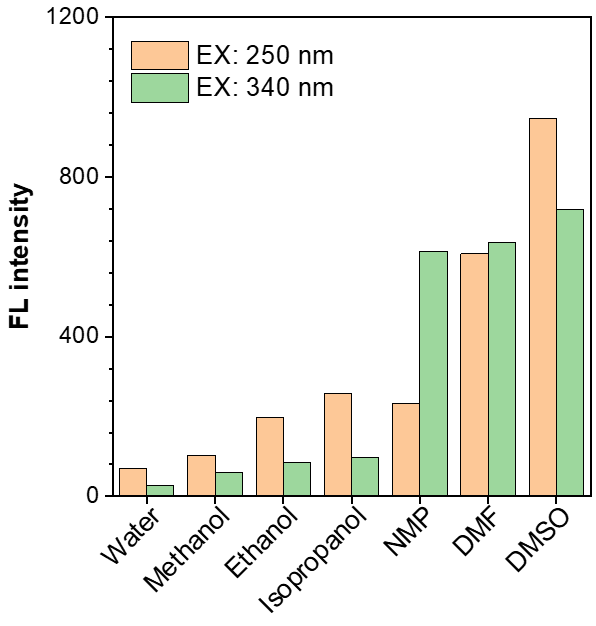


**Figure S10.** FL intensities of NCs-300 in protic and aprotic solvents. Compared with protic solvents, the FL intensity of NCs-300 in aprotic solvents can be increased by up to 25.5 times.


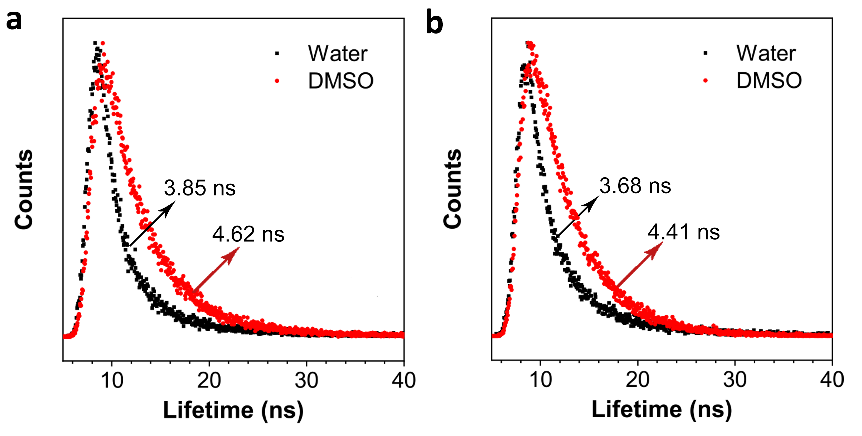


**Figure S11.** FL lifetimes of NCs-300 in water and DMSO measured by (a) 280 nm and (b) 375 nm laser. Results show that the lifetime of NCs-300 in DMSO is obviously higher than that of NCs-300 in aqueous solution.





**Figure S12.** Time stability of NCs-300 in aqueous solution measured at 250 nm and 340 nm. Results show that the FL intensity of NCs-300 decreases slightly in the initial stage and then remains constant as the storage time increases, indicating that NCs will not self-degrade in aqueous solution.





**Figure S13.** Photostability of NCs-300. EX: 340 nm. Results show that the obtained NCs display a strong photobleaching resistance, and their FL intensity is almost unchanged under 60 mins of illumination.





**Figure S14.** The FL intensity of NCs-300 at different pH solutions. EX: 340 nm. Results show that the FL efficiency of NCs can be greatly improved under acid solution, conversely, their FL intensity can be decreased with the increase of NaOH concentrations.





**Figure S15.** FL spectra of NCs-300 excited at 340 nm. Results show the pH-controlled reversible FL change of NCs-300.


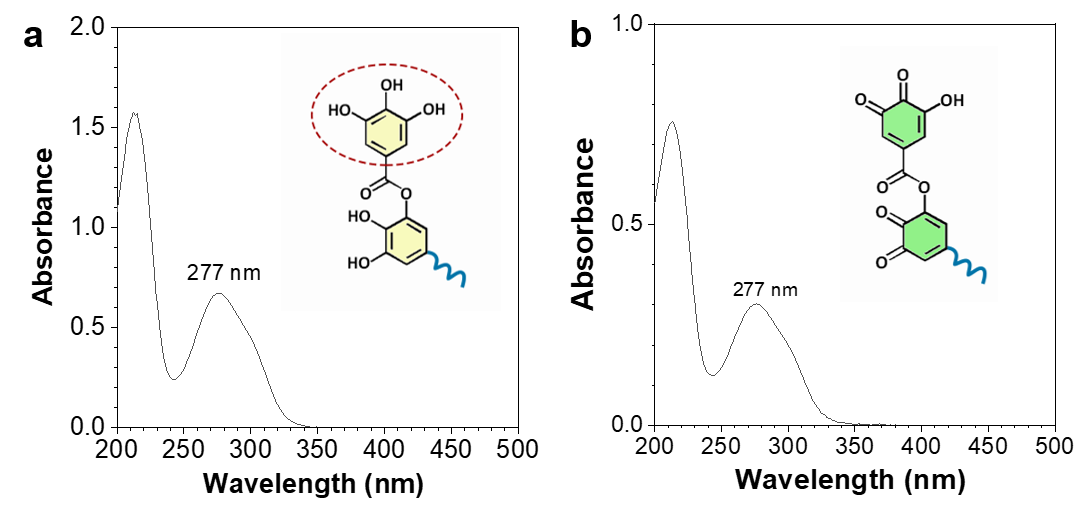


**Figure S16.** Absorption spectra of (a) TA and (b) oxidized TA. TA (0.1 g) is oxidized with H_2_O_2_ solution (5 mL, 3%) for 1 day at least to obtain oxidized TA. TA molecule shows a characteristic absorption peak at 277 nm because of its phenol structure.^[^[^2^](#_ENREF_2)^]^ After the formation of oxidized TA, the absorption peak has not obvious change.

**References**

[1] W. Yan, M. Shi, C. Dong, L. Liu, C. Gao, *Adv. Colloid Interface Sci.* **2020**, *284*, 102267.

[2] S. Quideau, D. Deffieux, C. Douat-Casassus, L. Pouységu, *Angew. Chem. Int. Ed.* **2011**, *50*, 586-621.
